# Persistent PR segment change in malignant pericardial disease

**DOI:** 10.1186/s40959-016-0015-1

**Published:** 2016-07-14

**Authors:** M. Ahluwalia, R. O’Quinn, B. Ky, D. Callans, J. Kucharczuk, J. R. Carver

**Affiliations:** 1 Department of Medicine, University of Pennsylvania Perelman School of Medicine, Hospital of the University of Pennsylvania, 3400 Spruce Street, 100 Centrex, Philadelphia, PA 19104 USA; 2 Department of Cardiovascular Medicine, University of Pennsylvania Perelman School of Medicine, Philadelphia, PA USA; 3 Department of Thoracic Surgery, University of Pennsylvania Perelman School of Medicine, Philadelphia, PA USA; 4 Abramson Cancer Center of the University of Pennsylvania, Philadelphia, PA USA

**Keywords:** PR segment, PR depression, PR elevation, malignant pericardial disease

## Abstract

**Background:**

Electrocardiographic changes may manifest in patients with pericardial effusions. PR segment changes are frequently overlooked, but when present, can provide diagnostic significance. The diagnostic value of PR segment changes in determining benign versus malignant pericardial disease in cancer patients with pericardial effusions has not been investigated. We aimed to determine the relationship between PR segment changes and malignant pericardial disease in cancer patients presenting with pericardial effusions.

**Methods:**

Consecutive patients with active malignancy who underwent surgical subxiphoid pericardial window by a single thoracic surgeon between 2011 and 2014 were included in this study. A total of 104 pre- and post-operative ECGs were reviewed, and PR depression or elevation was defined by deviation of at least 0.5 millivolts from the TP segment using a magnifying glass. Pericardial fluid cytology, flow cytometry and tissue biopsy were evaluated. Baseline characteristics and co-morbidities were compared between cancer patients with benign and malignant pericardial effusions.

**Results:**

A total of 26 patients with active malignancy and pericardial effusion who underwent pericardial window over the study period were included. Eighteen (69 %) patients had isoelectric PR segments, of whom none (0 %) had evidence of malignant pericardial disease (100 % negative predictive value). Eight (31 %) patients had significant ECG findings (PR segment depression in leads II, III and/or aVF as well as PR elevation in aVR/V1), all 8 (100 %) of whom had pathologically confirmed malignant pericardial disease (100 % positive predictive value). PR segment changes in all 8 patients persisted (up to 11 months) on post-operative serial ECGs. The PR segment changes had no relationship to heart rate or the time of atrial-ventricular conduction.

**Conclusions:**

In patients with active cancer presenting with pericardial effusion, the presence of PR segment changes is highly predictive of active malignant pericardial disease. When present, PR changes typically persist on serial ECGs even after pericardial window.

## Background

Malignant involvement of the pericardium has been detected in up to 20 % of patients diagnosed with cancer [1, 2], and can manifest as pericarditis, pericardial effusion, cardiac tamponade or constrictive pericarditis. The electrocardiogram (ECG) and radiologic imaging studies are noninvasive and may be helpful when evaluating pericardial effusions, but have low sensitivity and specificity for predicting the underlying etiology. Radiologic imaging studies, pericardiocentesis with fluid cytology analysis, and surgical pericardial biopsy may be utilized for diagnostic evaluation. Among these, the ECG is the most inexpensive, efficient test that serves clinical significance.

The PR interval is the electrical marker of atrial-ventricular (A-V) conduction time. Classically, transient PR depression associated with ST segment elevation can be seen in acute pericarditis and may further indicate the diagnosis when present. Persistent PR depression has been described in chronic inflammatory conditions [3–5]. PR segment changes have been correlated with atrial ischemia, larger territory infarct, post-myocardial infarction pericarditis and in the acute setting, may be associated with increased morbidity and overall poor prognosis [6–10].

In our clinical experience, we have observed persistent PR segment changes in cancer patients presenting with symptomatic or asymptomatic (incidentally diagnosed) pericardial effusions detected with transthoracic echocardiogram (TTE) or computed tomography (CT) scanning of the chest. Since the diagnostic value of PR segment changes in cancer patients presenting with pericardial effusion remains unknown, we aimed to analyze whether the presence or absence of significant PR segment changes in these patients is predictive of an underlying malignant etiology of the effusion.

## Methods

A consecutive study of 26 patients with active malignancy and pericardial effusion who underwent surgical subxiphoid pericardial window by a single thoracic surgeon between 2011 and 2014 was performed. A total of 104 pre- and post-operative paper ECGs were reviewed, and PR depression was defined by deviation of at least 0.5 milli-volts from the TP segment using a magnifying glass [3]. Two cardiologists independently confirmed the PR segment changes, and were blinded to the results of the biopsy. Age, gender, heart rate, indication for pericardial window, pericardial biopsy results, cytology and comorbidities including malignancy, cardiopulmonary disease and chronic non-cancer inflammatory disease were documented. Patients with persistent atrial fibrillation/flutter, acute pericarditis or connective tissue disorders were excluded.

Categorical variables were summarized by frequencies and proportions, and continuous variables were summarized by means and standard deviations or medians and interquartile ranges. T-tests were used to compare continuous variables with a normal distribution, Wilcoxon rank sum tests were used to compare continuous variables with a non-normal distribution, and Fisher’s exact tests were used to compare categorical variables. A *p*-value <0.05 was considered statistically significant.

## Results

**Fig. 1:**
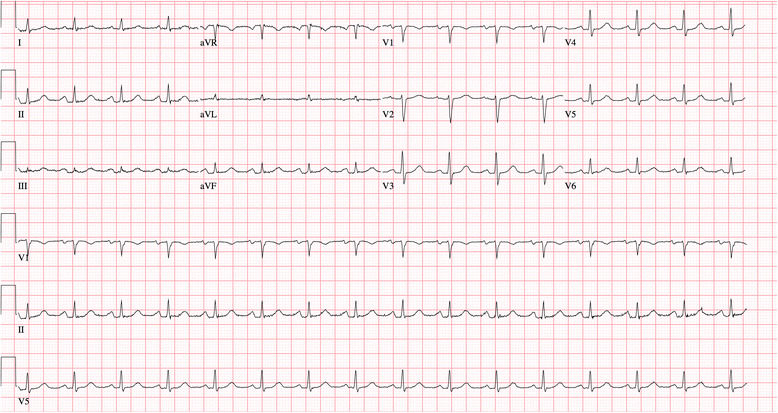
Normal sinus rhythm with isoelectric PR segments

**Fig. 2:**
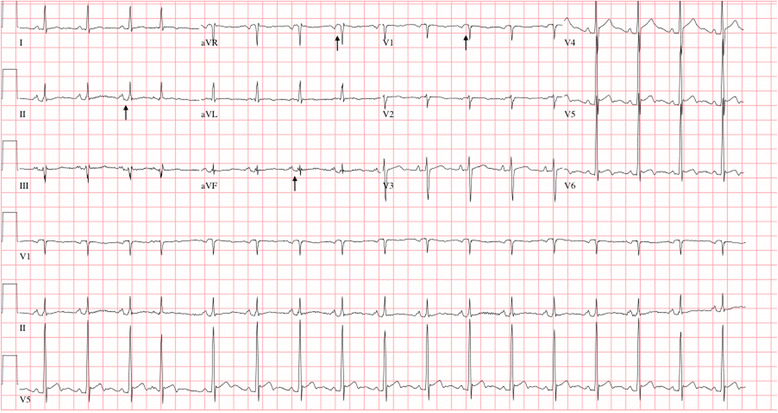
Electrocardiogram reveals sinus tachycardia, PR depression in leads II/avF and PR elevation in aVR/V1

A total of 26 cancer patients had pericardial windows placed from 2011 to 2014 by a single thoracic surgeon, and a total of 104 pre- and post-operative paper ECGs were reviewed. PR depression was defined by deviation of at least 0.5 milli-volts from the TP segment using a magnifying glass ^1^, which was confirmed by two cardiologists who independently confirmed the PR segment changes, and were blinded to the results of the biopsy. Eighteen (69 %) patients had isoelectric PR segments, of whom none (0 %) had evidence of malignant pericardial disease (100 % negative predictive value) as demonstrated by negative pericardial biopsy. Eight (31 %) patients had significant ECG findings (PR segment depression in leads II, III and/or aVF as well as PR elevation in aVR/V1), all 8 (100 %) of whom had pathologically confirmed malignant pericardial disease (100 % positive predictive value). PR segment changes were observed on pre- and post-operative serial ECGs without ST elevations. In each, the PR segment changes persisted up to 11 months on serial post-operative ECGs noted during long-term follow-up. Among the 8 patients identified with malignant pericardial effusion and positive ECG findings, one illustrative patient is described who is a 45 year-old male with metastatic head and neck squamous cell carcinoma who initially presented with shortness of breath and was found to have recurrent pericardial effusion. He underwent pericardial window. During follow-up visits, cardiovascular exam was notable for normal heart sounds without a rub. Pre- and post-operative electrocardiograms (ECG) (up to 7 months during follow-up) revealed persistent PR depression predominantly in leads II/aVF and PR elevation in aVR/V1 with a heart rate of 103 beats per minute (Fig. 1). Comparatively, an ECG is shown in Fig. 2 revealing normal sinus rhythm with isoelectric PR segments in a patient without malignant pericardial disease. All patients included had no evidence of previously described causes of PR segment change: acute ischemia, symptomatic pericarditis, systemic inflammatory disease or post-pericardiotomy syndrome. Baseline characteristics and co-morbidities are also compared between cancer patients with non-malignant and malignant pericardial effusions (Table 1).

**Table 1:** Baseline characteristics in cancer patients who underwent pericardial window for non-malignant and malignant pericardial effusions

	Non-malignant effusion, *n* = 18 (%)	Malignant effusion, *n* = 8 (%)	*p*-value
Age (years)	57.94 [19, 71]	54 [30, 69]	0.27
Male	11 (61.1)	3 (37.5)	0.40
Malignancy			
Lung	8 (44.4)	5 (62.5)	0.67
Breast	1 (5.56)	1 (12.5)	0.53
Leukemia	5 (27.8)	0 (0)	0.28
Hodgkin’s	3 (16.7)	0 (0)	0.53
Head & Neck	0 (0)	1 (12.5)	0.31
Myelodysplastic Syndrome	1 (5.56)	0 (0)	1.00
Other	2 (11.1)	1 (12.5)	1.00
Treatment			
Chemotherapy	16 (88.9)	7 (87.5)	1.00
Chemotherapy +	6 (33.3)	1 (12.5)	0.37
Chest Radiation			
Bone Marrow Transplant	3 (16.7)	0 (0)	0.53
Co-morbidities			
Hypertension	8 (44.4)	2 (25)	1.00
Dyslipidemia	7 (38.9)	1 (12.5)	0.36
Coronary Artery Disease	2 (11.1)	0 (0)	1.00
Pulmonary Embolism	3 (16.7)	3 (37.5)	0.33
Diabetes Mellitus	7 (38.9)	0 (0)	0.06
Paroxysmal Atrial Fibrillation	7 (38.9)	1 (12.5)	0.36
History of myopericarditis	1 (5.56)	3 (37.5)	0.07
Chronic obstructive pulmonary disease	3 (16.7)	1 (12.5)	1.00
Sepsis	2 (11.1)	0 (0)	1.00
Connective Tissue Disease	1 (5.56)	1 (12.5)	0.53
Chronic Kidney Disease	1 (5.56)	0 (0)	1.00
Other infection	1 (5.56)	0 (0)	1.00
Indication			1.00
Cardiac tamponade	10 (55.6)	7 (87.5)	0.19
Impending cardiac tamponade	8 (44.4)	1 (12.5)	0.19
Pericardial effusion	3 (16.7)	0 (0)	0.53
Heart rate (beats per minute)	101 [76, 128]	108 [88, 128]	0.25
ECG			<0.001
Positive*	0 (0)	8 (100)	
Negative**	18 (100)	0 (0)	
Pathology			<0.001
Positive	0 (0)	8 (100)	
Negative	18 (100)	0 (0)	

Baseline characteristics including age, sex, underlying malignancy, treatment course, co-morbidities, indication for pericardial window, heart rates calculated by ECGs, ECG findings and pericardial pathology are compared between cancer patients with non-malignant and malignant pericardial disease. *Positive ECG findings are defined as PR depression in leads I, II and/or aVF and PR elevation in aVR/V1. **Negative ECG findings are defined as isoelectric PR segments. Positive pathology is defined as positive pericardial fluid cytology, biopsy and/or flow cytometry for malignancy. T-tests were used to compare continuous variables with a normal distribution, Wilcoxon rank sum tests were used to compare continuous variables with a non-normal distribution, and Fisher’s exact tests were used to compare categorical variables. A *p*-value <0.05 was considered statistically significant

## Discussion

Malignant pericardial effusions are most commonly seen in active solid tumors such as breast and lung cancer, and hematologic malignancies. ECG evaluation for PR segment changes is often ignored, but it is an important tool for diagnostic evaluation, especially when used in the appropriate clinical setting. This study adds an additional dimension to prior observations of PR segment changes, since analysis of PR segment depression has not been previously implicated as a marker for malignant pericardial disease. PR segment depression was predominantly seen in leads II, III and/or aVF. PR depression was horizontal or concave in morphology. Additionally, convex PR elevation in aVR and/or V1 was observed in combination with PR depression in the inferior leads. The PR segment changes had no relationship to heart rate or the time of A-V conduction. Patients did not have symptoms of acute ischemia, systemic inflammatory disease, myopericarditis or post-pericardiotomy syndrome that could otherwise explain PR segment changes.

The mechanism of PR segment changes described in malignant pericardial effusion may be the result of either direct invasion of the pericardium, or hematogenous versus lymphangitic spread that could potentially affect atrial repolarization. This may then manifest as PR segment depression or elevation in the leads representing the site of malignant pericardial involvement. In addition, patients often undergo chemotherapy and/or radiation, which may result in inflammation and abnormal conduction. Infections can also promote an inflammatory state. A recent study observed clinical correlates of transient PR segment depression in asymptomatic pericardial effusions [3]. In this sub-group, patients had underlying post-pericardiotomy syndrome, systolic heart failure or connective tissue disease, and 40 % had malignant effusions [3]. This suggests that one likely pathogenesis of PR segment changes is inflammatory in nature. The finding of persistent ECG changes as presented in this case series may infer that the malignant involvement is a sustained process, unlike pericarditis and post-pericardiotomy syndrome, which often respond to anti-inflammatory therapy.

This study has several limitations. First, this is a descriptive report of a new finding in a small number of patients who underwent pericardial window by one thoracic surgeon at a single institution. Although we obtained 100 % sensitivity and specificity in this observational case series, PR segment changes cannot be regarded as specific for malignant pericardial effusion. A larger prospective would be helpful to further determine the relationship between PR segment change and advanced malignant disease, and subsequent treatment of the malignancy as a marker of treatment success or failure.

We propose that evidence of PR segment changes on serial ECGs in the absence of myopericarditis, post-pericardiotomy syndrome or other systemic inflammatory disease should raise suspicion for malignant pericardial disease, and thus can provide diagnostic significance. Overall, surveillance ECGs may be cost-effective without additional risk compared to surveillance radiographic imaging to determine the status of tumor progression.

## Conclusions

PR segment changes in cancer patients are likely more prevalent in patients with malignant pericardial disease and may provide diagnostic value in a cancer patient who presents with pericardial effusion.

## Abbreviations

A-V conduction, Atrial-ventricular conduction; CT, Computed tomography; ECG, Electrocardiogram; TTE, Transthoracic echocardiogram
